# Supplementary material for: ON-OFF receptive fields in auditory cortex diverge during development and contribute to directional sweep selectivity
Source: Nat Commun. 2018 May 25;9:2084. doi: 10.1038/s41467-018-04548-3 (PMC5970219; doi:10.1038/s41467-018-04548-3)
Supplement: Supplementary file 1 — Supplementary Information [file 41467_2018_4548_MOESM1_ESM.pdf]

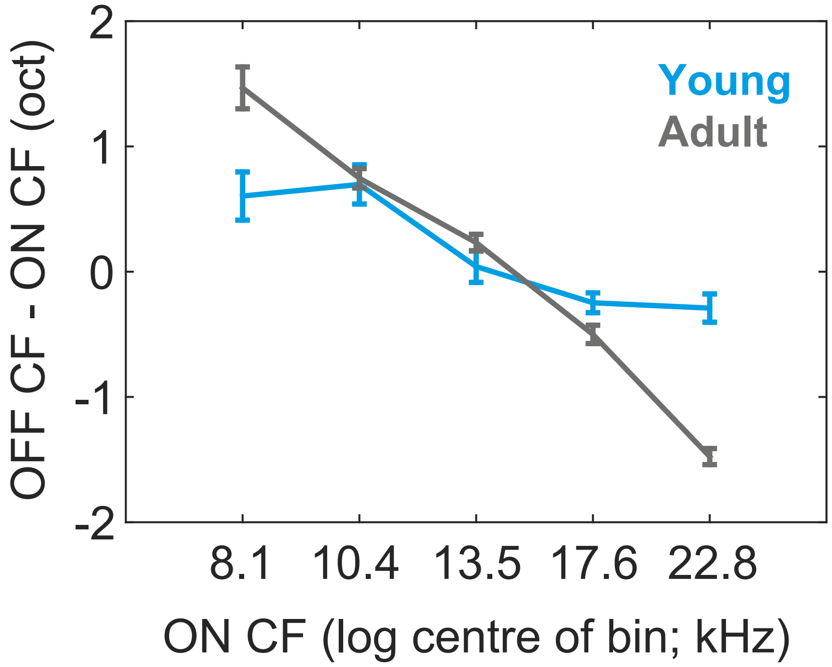

Supplementary Figure 1 Systematic bias of ON/OFF CF difference with respect to ON CF in young and adult A1 recordings.

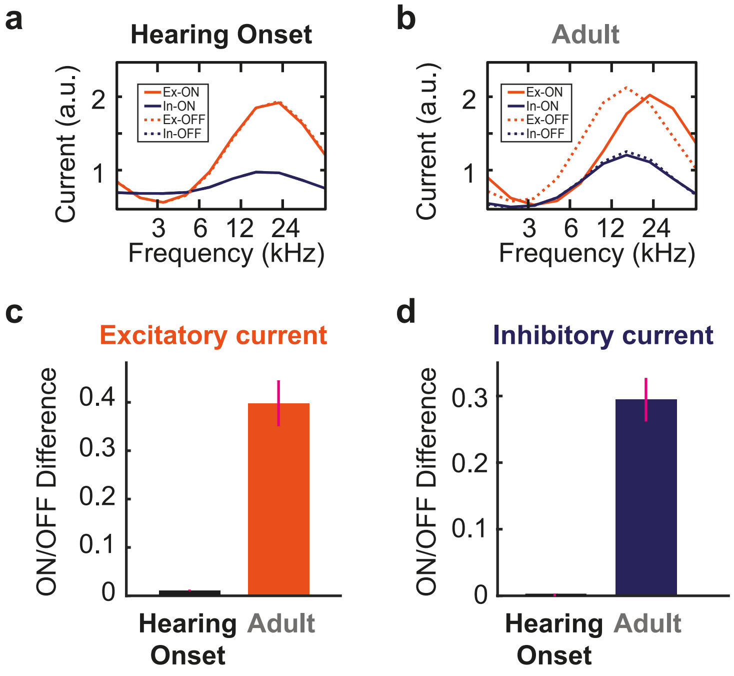

Supplementary Figure 2 Divergence of ON and OFF current selectivity via Hebbian learning. **a**, Example frequency selectivity for synaptic currents at beginning of simulation (Hearing Onset). **b**, Example frequency selectivity for synaptic currents at end of simulation (Adult). **c,d**, Mean absolute difference between ON and OFF peak selectivity of excitatory (**c**) and inhibitory (**d**) synaptic currents at beginning and end of the simulation (Hearing Onset and Adult respectively).

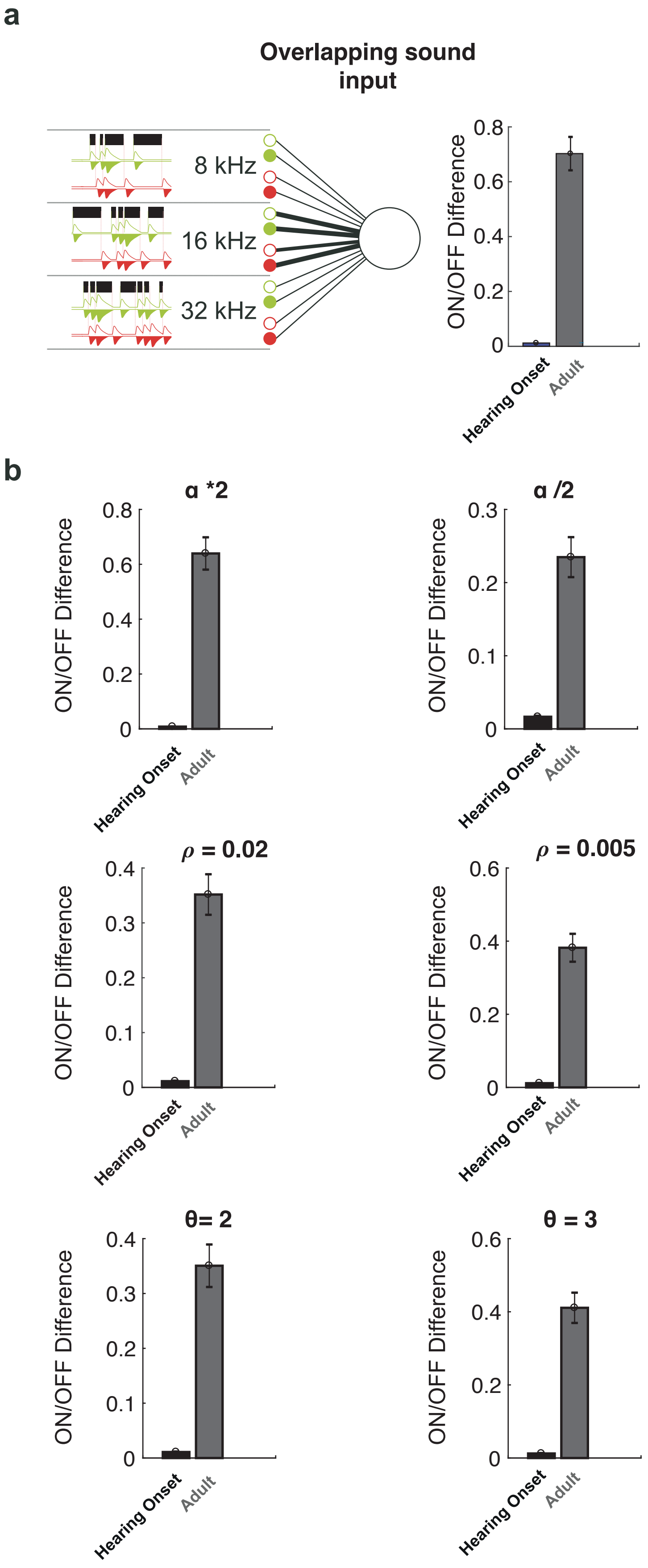

Supplementary Figure 3 Robustness of ON/OFF CF divergence to (**a**) overlapping sound input and (**b**) model parameters (See Methods for further information).

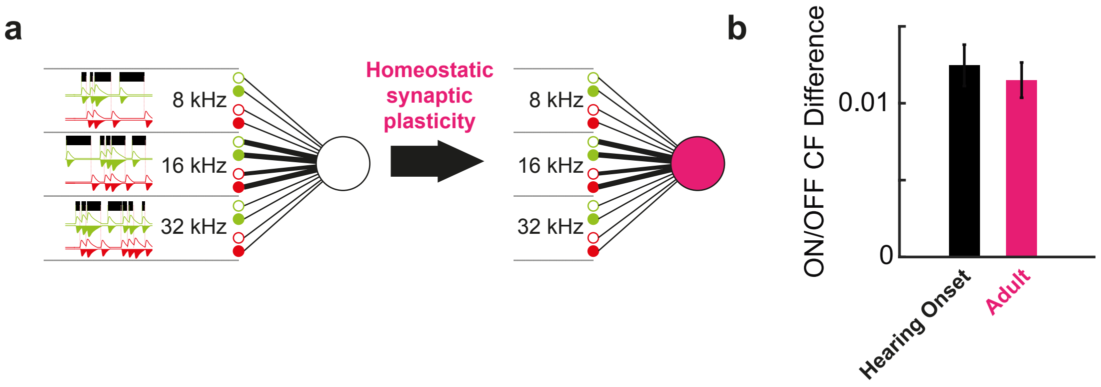

Supplementary Figure 4 Homeostatic plasticity alone cannot account for developmental divergence of ON and OFF RFs. **a**, Simulated network is comprised of a neuron (white; left) that receives 10 excitatory and 10 inhibitory ON inputs (green, open and filled circles respectively), and 10 excitatory and 10 inhibitory OFF inputs (red, open and filled circles respectively). Only 3 ON and OFF input channels are shown for clarity. Synaptic weights undergo plasticity during sound presentation according to a homeostatic plasticity rule. At the end of the simulation (adult condition in magenta, right), ON/OFF synaptic weights have not diverged. **b**, Mean absolute difference between ON and OFF CF at Hearing Onset (black) and Adult (magenta) neurons (n = 100). Note the very small absolute values for CF difference in comparison to Hebbian learning model (**Figure 2**).

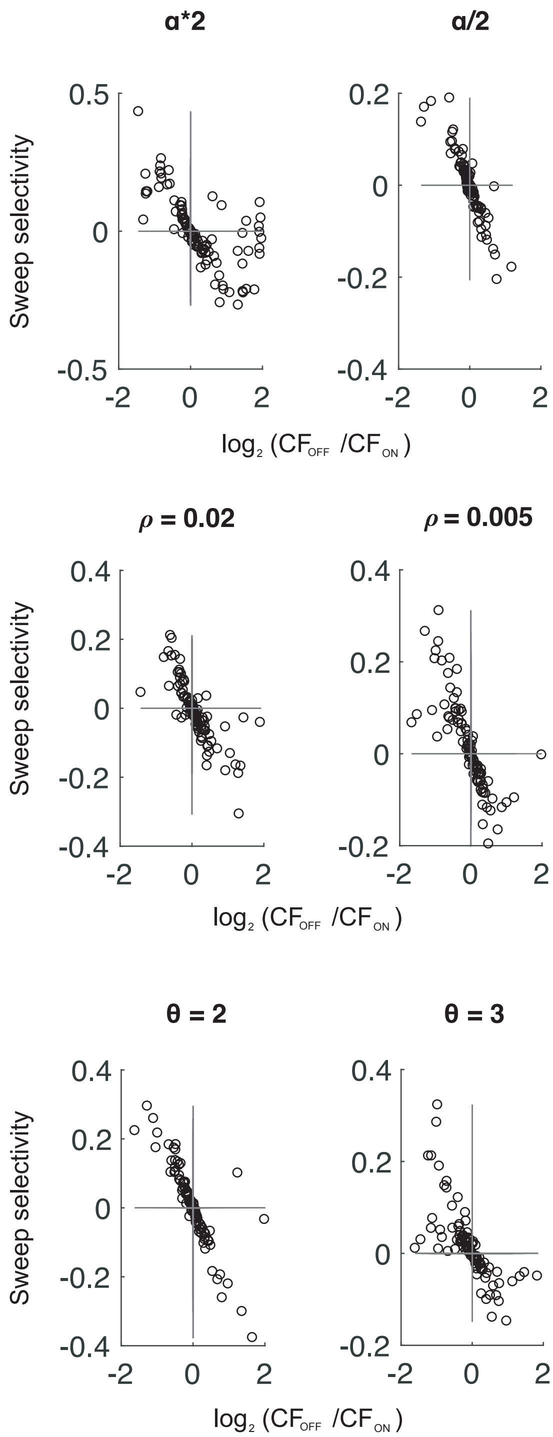

Supplementary Figure 5 Robustness of sweep selectivity to model parameters (See Methods for further information).

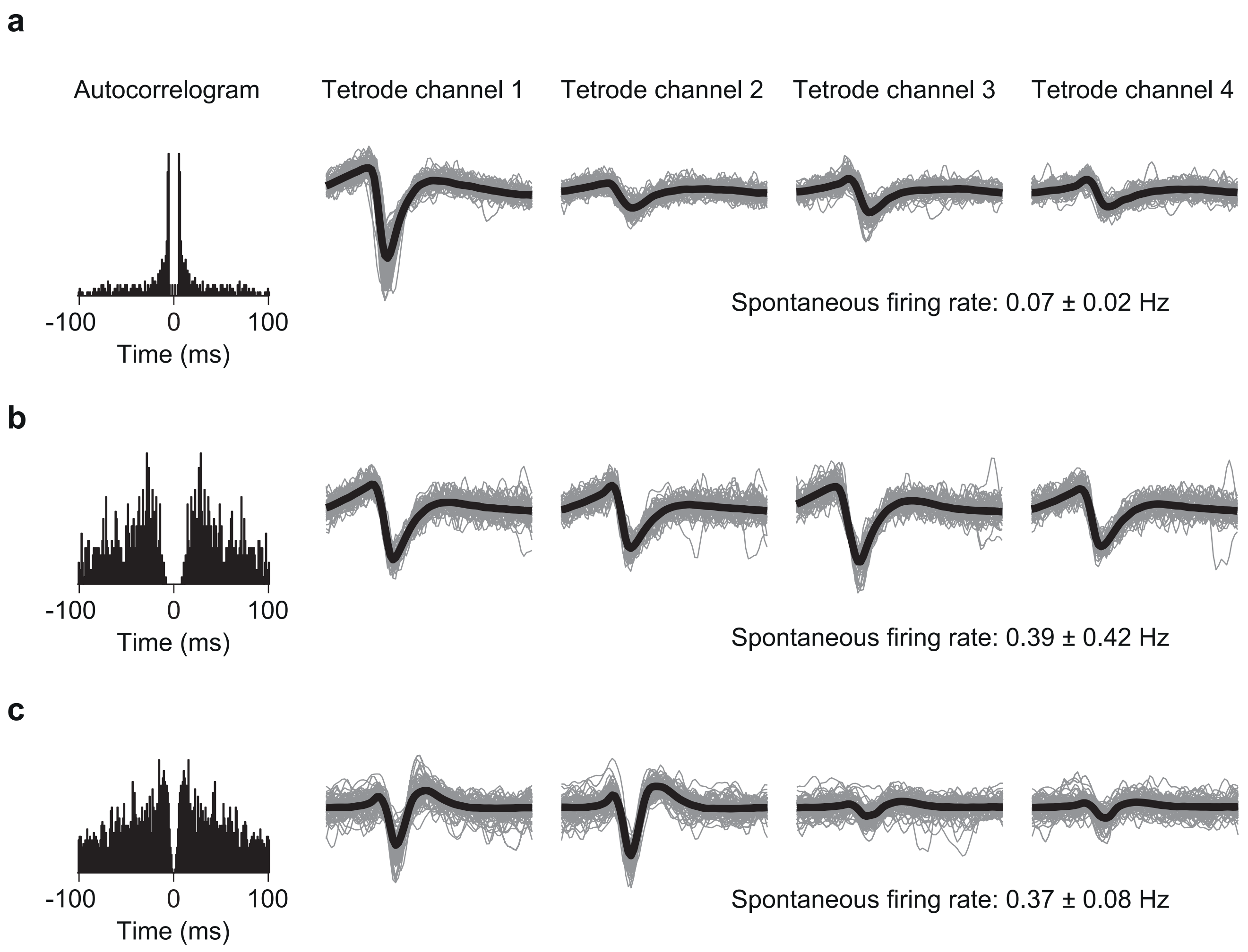

Supplementary Figure 6 Example single units recordings to quantify ON-OFF CF difference and DSI *in vivo*. Autocorrelograms (left) and spike waveforms (right; 50 overlaid traces, mean in black) for single units shown in Figure 5a (**a**), Figure 5b (**b**), and Figure 6i (**c.**)

Supplementary Tables 1-3 Assessing contribution of functional neuronal properties to slow direction selectivity in A1

I) Adult data

To test the contribution of different neural properties in predicting the DSI, we generated linear multi-variable models based on the cell properties recorded in adult mice. The importance of each property in predicting DSI was assessed via two parameters, namely the absolute value of the normalised coefficient, and the proportional reduction of error (PRE) generated when adding the property as a predictor into the model (see also Materials and Methods). The performance of the model in predicting DSI was assessed via the adjusted R-square, which takes into account the number of parameters used.

First, we built a model encompassing all neural properties judged relevant (n = 10 properties, namely the firing rate (FR) of the ON response to tones of CF_{on} frequency at 60dB, FR of the OFF response to tones of CF_{off} frequency at 60dB, Fano factor (FF) of each of these two responses, spontaneous firing rate and associated FF, bandwidths (BWs) of ON and OFF RFs (measured at 30dB), percentage overlap between ON and OFF RFs, and octave difference between ON and OFF CFs). This model was called the **Full** model. The adjusted R-square of the model was 0.279, which was worse than the adjusted R-square of the single-variable model built using ON/OFF CF difference as sole predictor (adjusyed R-square = 0.348). It is important to highlight that in the Full model, ON/OFF CF difference had the largest coefficient value (**Supplementary Table 1**), and that it accounted for a large portion of the error reduction.

**Prop.**	FR_{ON}	FR_{OFF}	FF_{ON}	FF_{OFF}	FR_{spont}	FF_{spont}	BW_{ON}	BW_{OFF}	ON/OFF overlap	ON/OFF CF diff.
**Coeff.**	-0.021	0.007	-0.012	0.001	0.025	0.002	-0.090	0.151	-0.194	-0.355
**PRE**	1.257	0.100	0.365	0.002	0.100	0.009	0.733	2.536	0.774	0.907

Supplementary Table 1: Normalised coefficients and proportional reduction of error for the **Full** model. PRE for variables considered highly predictive are marked in yellow. Adj. R-square 0.279.

The lower R-square in the Full model could originate from spurious variables which do not add any predictive information. By iteratively adding and removing variables from the Full model, the **Optimal** model was found to be composed of the following predictors: the firing rate of the ON response to tones of CF_{on} frequency at 60dB, the bandwidth of the OFF RF, the percentage overlap between ON and OFF RFs, and the octave difference between ON and OFF CFs. The coefficients and PRE for the predictors of this model are presented in **Supplementary Table 2**. This model had an adjusted R-square of 0.419. Any addition or removal of predictor to this model decreased the adjusted R-square.

**Prop.**	FR_{ON}	FR_{OFF}	FF_{ON}	FF_{OFF}	FR_{spont}	FF_{spont}	BW_{ON}	BW_{OFF}	ON/OFF overlap	ON/OFF CF diff.
**Coeff.**	-0.012	/	/	/	/	/	/	0.123	-0.169	-0.323
**PRE**	0.380	/	/	/	/	/	/	1.526	0.517	0.691

Supplementary Table 2: Normalised coefficients and proportional reduction of error for the **Optimal** model. PRE for variables considered highly predictive are marked in yellow. Adj. R-square 0.419.

II) Young data

To further show that the properties found in Young neurons could not account for the DSI, we developed a Full linear multi-variable model (using the same parameters as for Adult neurons). In the case of Young neurons, the difference between ON-OFF CFs was not a good predictor of the DSI (**Supplementary Table 3**). Instead, the percentage overlap was found to be the variable with the best PRE. However, the percentage overlap was not a good predictor of DSI when used as single-variable model (adjusted R-square 0.059). We thus conclude that there was no clear linear relationship between DSI and any of the tested neuronal properties in Young mice.

**Prop.**	FR_{ON}	FR_{OFF}	FF_{ON}	FF_{OFF}	FR_{spont}	FF_{spont}	BW_{ON}	BW_{OFF}	ON/OFF overlap	ON/OFF CF diff.
**Coeff.**	-0.004	0.008	0.010	-0.028	0.207	0.025	-0.120	0.066	-0.638	-0.151
**PRE**	0.008	0.015	0.055	0.956	0.493	1.080	1.111	0.173	4.370	0.077

Supplementary Table 3: Normalised coefficients and proportional reduction of error for the **Full** model (Young). PRE for variables considered highly predictive are marked in yellow. Adj. R-square -0.339.
